# Potential Benefits of Dietary Fibre Intervention in Inflammatory Bowel Disease

**DOI:** 10.3390/ijms17060919

**Published:** 2016-06-14

**Authors:** Celestine Wong, Philip J. Harris, Lynnette R. Ferguson

**Affiliations:** 1 Auckland Cancer Society Research Centre, Faculty of Medical and Health Sciences, The University of Auckland, Private Bag 92019, Auckland 1142, New Zealand; cwon826@aucklanduni.ac.nz; 2 School of Biological Sciences, The University of Auckland, Private Bag 92019, Auckland 1142, New Zealand; p.harris@auckland.ac.nz; 3 Discipline of Nutrition and Dietetics, Faculty of Medical and Health Sciences, The University of Auckland, Private Bag 92019, Auckland 1142, New Zealand

**Keywords:** dietary fibres, human intervention, inflammatory bowel disease, Crohn’s disease, ulcerative colitis

## Abstract

Intestinal dysbiosis is thought to be an important cause of disease progression and the gastrointestinal symptoms experienced in patients with inflammatory bowel disease (IBD). Inflammation appears to be a major contributor in perpetuating a dysregulated gut microbiota. Although current drug therapies can significantly induce and maintain disease remission, there is no cure for these diseases. Nevertheless, ongoing human studies investigating dietary fibre interventions may potentially prove to exert beneficial outcomes for IBD. Postulated mechanisms include direct interactions with the gut mucosa through immunomodulation, or indirectly through the microbiome. Component species of the microbiome may degrade dietary-fibre polysaccharides and ferment the products to form short-chain fatty acids such as butyrate. Prebiotic dietary fibres may also act more directly by altering the composition of the microbiome. Longer term benefits in reducing the risk of more aggressive disease or colorectal cancer may require other dietary fibre sources such as wheat bran or psyllium. By critically examining clinical trials that have used dietary fibre supplements or dietary patterns containing specific types or amounts of dietary fibres, it may be possible to assess whether varying the intake of specific dietary fibres may offer an efficient treatment for IBD patients.

## 1. Introduction

Inflammatory bowel disease (IBD) are a group of gastrointestinal disorders characterised by chronic inflammation and dysbiosis of the gut microbiota [1,2]. Two major forms of IBD are Crohn’s disease (CD), which may affect the small and/or large intestine, the mouth, oesophagus, stomach and anus, and ulcerative colitis (UC), which affects primarily the colonic mucosa. Although CD has been traditionally defined as a chronic inflammatory condition that can be located anywhere on the epithelial layer of the gastrointestinal tract (GIT) [3,4], a recent analysis suggests that CD might itself be divided into two classes, depending partly on the disease location [5]. Clinical features that all IBD patients commonly experience include diarrhoea and/or constipation, and abdominal cramping following bowel movements. Furthermore, IBD complications that can arise are an obstructed bowel and the passing of mucus, pus or blood [6,7].

Although pharmaceutical approaches to disease control have improved in recent years, there is still a high frequency of patients who are refractory to current drugs, including both conventional and biological therapies [8]. Besides the role of genetic predisposition in the development of IBD [9], diet and nutritional components have been considered important in the course of the disease, especially where this is early onset [10]. It is possible for dietary modifications to either help induce disease remission or exacerbate adverse symptoms by influencing disease activity, and/or altering the gut mucosal immune system [11,12]. Therefore, it has been suggested that nutritional therapy can be aimed at reducing gut inflammation and consequently helping to prevent relapse [13,14,15]. Nutritional therapy may also act indirectly, through effects on the colonic microbiome [16]. The human intestinal microbiome is composed of a diverse group of microorganisms colonizing the gastrointestinal (GI) tract. When functioning optimally, this is actively involved in nutrient metabolism, energy homeostasis and regulates immune responses, protecting the GI tract from harmful dietary pathogens [17,18]. A eubiotic gut microbiota is characteristic of healthy individuals. It is defined by a predominance of potentially beneficial species being found in stool samples, primarily belonging to Firmicutes and Bacteriodes, but usually also with minor amounts of Protobacteria and Actinobacteria [19,20]. Its converse, intestinal dysbiosis, is associated with an imbalance of this microbial composition, often associated with excessive numbers of adverse enterobacteria and Gram-negative bacteria as compared with beneficial microbes [19,20,21].

The possible roles of dietary fibres (DFs) in the etiology of CD and UC are unclear. Diets low in DF (low residue diets) are sometimes recommended to patients with the active forms of the diseases [22], while recommendations for patients with the inactive forms have usually not considered dietary fibre, partly because individuals differ in dietary tolerances and intolerances [23,24]. Different types of DF have different properties and health effects in diseases such as IBD. Potential benefits include reducing diarrhoea or constipation, producing short-chain fatty acids (SCFAs), down-regulating inflammation, promoting tissue healing, and by these means potentially preventing the onset of colorectal cancer (CRC) in susceptible IBD patients [25,26,27,28,29].

In this review, we initially discuss ways of evaluating IBD and also current understandings on the nature of dietary fibre. The findings of studies in the literature that investigated the effects of DF in IBD patients are examined to determine possible associations between DF intake and subsequent alterations to disease symptoms. Some possible mechanisms including changes to the impaired gut microbiota, as well as more direct mechanisms, are considered. In addition, the methodology and endpoint measures used to assess potential benefits for IBD are evaluated.

## 2. Results

### 2.1. Assessments of Inflammatory Bowel Disease (IBD)

The assessment of IBD activity may be subdivided into measures of disease activity and measures of the impact on quality of life. Studies conducted before the new millennium used few methodological tools that could quantitatively measure the outcomes of DF intervention. Investigators used general questionnaires, the frequency of clinical hospital admissions and surgeries, ileostomy fluid output, and clinical, endoscopic or symptomatic scores [30,31,32,33,34]. Endoscopy is a diagnostic or therapeutic procedure whereby a thin flexible endoscope is used to view the lining of the bowel, while colonoscopy is an endoscopy of the large bowel (colon) [35]. Endoscopy is an essential tool for diagnosing and treating both CD and UC. It allows an initial diagnosis, distinguishing CD from UC, assessing disease extent and activity, monitoring response to therapy and surveying for dysplasia [35]. Colonoscopic examination has also been used to determine the extent of disease progression in a number of studies [36]. However, endoscopy will not be sufficiently sensitive to detect a number of more subtle symptoms of the disease, especially those relating to quality of life.

More recent studies have exploited a wide range of tools to more accurately measure the impact of DF intervention on IBD patients. Overall, two types of analysis have been used. The first involved detailed symptomatic scoring methods with questionnaires, daily food diaries, and clinical disease activity scales. Examples include visual analogue scales [33] in which patients could rate the severity of their symptoms, and scores from clinical activity indices, including the Crohn’s disease activity index (CDAI) [2] and Harvey–Bradshaw index (HBI) [21,37], that can assess the extent of disease activity. It is apparent that CDAI scores are weighted based on symptoms experienced and a score of <150 implies clinical remission in patients with CD [38]. However, it is noteworthy that such self-evaluated scores do not correlate well with colonoscopic or endoscopic analysis [38]. The HBI is similar to the CDAI, with a remission score of <5 correlating to a CDAI score of <150. Furthermore, IBD questionnaires (IBDQ) offer investigators an indication of the patient’s quality of life following dietary intervention [4].

The second types of analyses used faecal or serum biomarkers that can determine the extent of changes following DF intervention. Biomarkers are defined as “measureable substances derived from a biofluid or tissue specimens” [39]. Two of these are commonly used as measures of inflammation in IBD. C-reactive protein (CRP) is measured from serum, and provides a general and very sensitive measure of systemic inflammation, albeit not specific to the GI tract (GIT) [40,41]. Erthrocyte sedimentation rate (ESR) is the other measure which is obtained from a blood sample. This is the rate at which red blood cells sediment in a period of one hour, providing a non-specific measure of inflammation [39]. Immune biomarkers have also been used as measures of inflammatory activity. For example, the concentrations of interleukin-10 (IL-10), which is an anti-inflammatory cytokine, can be measured in serum samples from patients and compared before and after intervention with DF [40]. Faecal assays are more likely to reflect inflammation at the mucosal surface, and faecal calprotectin (fcalpro) is increasingly used as a measure of the extent of GIT inflammation [42]. Other potential faecal biomarkers, albeit not formally recognized as such by regulatory bodies at present, include the bacterial composition, especially the proportion of certain bacterial species, such as *Bifidobacterium*, *Eubacterium limosum*, *Akkermansia muciniphila*, and *Clostridium* cluster XIVa species, which are present in the patient’s GIT [36,43]. The concentrations of faecal SCFAs such as butyrate have also been used as an endpoint [21,37]. Overall, the development of such modern approaches to assess the efficacy of dietary intervention in patients with IBD should provide investigators with more sensitive endpoints to infer associations between DF intake and disease remission. It should be noted however, that if dietary interventions are to be considered therapeutic, one would expect to see clear dose−effect relationships, alongside a pharmacodynamic explanation.

### 2.2. Structures and Compositions of Dietary Fibres

Following many years of debate, the Codex Alimentarius Commission [44] defined dietary fibre as follows:

“Dietary fibre means carbohydrate polymers (see details in (a) below) with ten or more monomeric units (see details in (b) below), which are not hydrolysed by the endogenous enzymes in the small intestines of humans and belong to the following categories: edible carbohydrate polymers naturally occurring in the food as consumed; carbohydrate polymers, which have been obtained from food raw material by physical, enzymatic or chemical means and which have been shown to have a physiological effect of benefit to health as demonstrated by generally accepted scientific evidence to competent authorities; and synthetic carbohydrate polymers which have been shown to have a physiological effect of benefit to health as demonstrated by generally accepted scientific evidence to competent authorities.

(a) When derived from a plant origin, dietary fibre may include fractions of lignin and/or other compounds associated with polysaccharides in plant cell walls. These compounds also may be measured by certain analytical method(s) for dietary fibre. However, such compounds are not included in the definition of dietary fibre if extracted and re-introduced into a food.

(b) Decision on whether to include carbohydrates from 3 to 9 monomeric units should be left to national authorities.”

Following the acceptance of the Codex Alimentarius Commission definition of DF [44], new methods for its quantification have been introduced, including a total DF method, which has been adopted as the AOAC Official Method 2009.01 [45], and a version of the method that measures both soluble and insoluble DF referred to as AOAC Official Method 2011.25 [46].

In diets containing few processed foods, much of the DF consists of the cell walls of food plants. These walls are all constructed similarly, with microfibrils of the polysaccharide cellulose set in a matrix of non-cellulosic polysaccharides, the types and proportions of which vary depending on plant species and cell type. The non-cellulosic polysaccharides include pectins (pectic polysaccharides) and hemicelluloses with a variety of different structures. In the cell walls of most fruits and vegetables, these polysaccharides are mainly pectins, with smaller proportions of the hemicellulose xyloglucan. In contrast, in the cell walls of cereal grains, hemicelluloses are predominant. The hemicellulose arabinoxylan, which has a 1,4-β-xylan backbone and arabinose side chains, predominates in wheat, whereas 1,3;1,4-β-glucan, which is a linear polymer of β-glucosyl units containing both 1,3- and 1,4-links, usually predominates in oats and barley cell walls. In addition, small proportions of lignin are present in the outer layers of some brans, particularly wheat bran [27,47,48,49]. Details in (a) of the above definition of DF specifies that any lignin present in the cell walls of food plants is included in the definition. Lignin is not a polysaccharide but an aromatic, hydrophobic polymer formed by the coupling of 4-hydroxycinnamyl alcohols. It often crosslinks cell-wall polysaccharides and impedes their enzymatic degradation [50]. Preparations rich in plant cell walls are often used as supplements. For example, bran preparations consist of the outer layers of cereal grains. Oat brans are rich in 1,3;1,4-β-glucans, a high proportion of which are water soluble [51]. In contrast, wheat brans contain high proportions of arabinoxylans, which are mostly water insoluble, and significant proportions of lignin [27]. A preparation rich in plant cell-wall components derived from malted barley after being used in beer making (brewer’s spent grains) has been used as a supplement. This is known as germinated barley foodstuff and is generated by wet grinding followed by sieving [52]. The preparation is rich in water-insoluble arabinoxylans and also contains lignin, presumably from small amounts of husk not removed in the sieving. It also has a high (46%) content of glutamine-rich protein [53].

In addition to intact plant cell walls, the definition of DF includes preparations of polysaccharides extracted from cell walls, such as pectin preparations, which are used in the food industry. The definition also includes polysaccharides that are not derived from plant cell walls. For example, the mucilage polysaccharides produced by the epidermis (outer layer) of the coats (husks) of some seeds, such as those of *Plantago ovata* (known as psyllium or ispaghula), and on wetting the mucilage polysaccharides swell to form a gel. In psyllium, the gel-forming polysaccharides are mostly highly substituted xylans (heteroxylans) [54]. Psyllium husks are frequently used as DF supplements, with both the mucilage polysaccharides and the cell walls of the husks being DFs.

The DF definition also includes resistant starch defined as “the sum of starch and products of starch degradation not absorbed in the small intestine of healthy individuals” [55]. Four types of resistant starch (RS1–4) are often recognized [47]. RS1 occurs in the form of raw (native) starch granules present in the cells of intact or coarsely milled grains or other starch-rich seeds. The starch is protected from degradation because of its physical inaccessibility to α-amylases. RS2 starch also occurs in the form of raw granules, but in this type, they are resistant to α-amylases because of their crystallinity. Examples of this type include the starch grains in green banana fruits, uncooked potatoes, and cultivars of plants containing high-amylose starch grains, such as the maize cultivar Hi-Maize™. RS3 starch is known as retrograded starch and is produced on cooling gelatinized starch, formed by heating raw starch granules in aqueous solutions, as occurs in cooking. It occurs in cooled cooked foods such as potatoes and bread. Of the two polysaccharides that comprise starch, amylose solutions retrograde on cooling more readily than amylopectin solutions do. High-amylose starches are thus particularly good at producing RS3. RS4 comprises starches that are chemically modified, by being, for example, esterified, etherized, or crosslinked. Such starches are commonly used in processed foods. The different forms of RS have different properties and may have different effects in CD and UC.

Although not degraded in the small intestine, DF carbohydrates may be degraded in the colon by bacterial enzymes and the products fermented by bacteria to produce short-chain fatty acids (SCFAs) (mainly acetic, butyric, and propionic acids), carbon dioxide, hydrogen, methane, and water. The proportions of the different SCFAs are determined by the bacterial species as well as the type of DF. Because degradative enzymes can easily access substrates in solution, if all the appropriate enzymes are present, it would be expected that soluble DFs will degrade quickly and extensively upon entering the colon. This has been demonstrated to occur with a soluble pectin preparation that was degraded almost fully and mostly in the proximal colon [56]. However, psyllium preparations are only partly degraded, presumably because not all the enzymes necessary for complete degradation are present. Thus, simply because a DF is soluble does not necessarily mean it will be quickly and fully degraded in the colon.

#### 2.2.1. Non-Digestible Oligosaccharides

Non-digestible oligosaccharides (NDOs) are another group of components often included in DF. Although the Codex Alimentarius Commission definition of DF specifies carbohydrate polymers with ten or more monomeric units, detail (b) (see above) concedes that carbohydrates with 3–9 monomeric units may also be included, with the decision left to national authorities. Since the publication of this definition, many jurisdictions have resolved to include these oligosaccharides in the definition [57]. Some NDOs occur naturally in food plants, whereas others are synthetic or semisynthetic. These water-soluble oligosaccharides are resistant to digestion in the small intestine, but are degraded by bacteria in the colon.

This group includes the fructans, which are molecules based on the monosaccharide fructose, and occur widely in plants, including garlic, onions and asparagus, with differences in the types of linkages and whether the molecules are linear or branched [58]. Inulin-type fructans are linear molecules with β-2,1-linkages and are commercially extracted from chicory roots. From this source, the molecules range in length from 2 to ~60 units, with an average degree of polymerization (DP) of 12. Thus, although usually regarded as oligosaccharides, technically the molecules range from oligosaccharides to small polysaccharides, with the molecules with DPs of 2–9 being referred to as oligofructoses (or fructo-oligosaccharides), and those with DPs of 10 and greater as high molecular weight inulin [59,60]. NDOs occurring naturally in food plants also include the α-galactosides, which are present particularly in the seeds of leguminous plants and include the trisaccharide raffinose, the tetrasaccharide stachyose, and the pentasaccharide verbascose [58].

#### 2.2.2. FODMAPs (Fermentable Oligo-, Di- and Mono-Saccharides, and Polyols)

Together with the monosaccharide fructose, the disaccharide lactose and polyols (sugar alcohols), NDOs form part of a group of low molecular weight carbohydrates known by the acronym FODMAPs: Fermentable oligo-, di- and mono-saccharides, and polyols [61]. These FODMAP carbohydrates are poorly absorbed in the small intestines for a variety of reasons, and because they are small molecules, they cause the osmotic uptake of water into the intestinal lumen. This speeds their movement into the large intestine where they are rapidly degraded and fermented [61]. In susceptible individuals on a diet with a high content of these carbohydrates, the increased volumes of liquid and gas in the intestines leads to bloating and abdominal pain. Following a low-FODMAP diet to suppress such adverse symptoms has been systematically developed to reduce these problems. However, it must be remembered that such diets also reduce the amount of DF in the form of NDOs, and these may themselves have longer term physiological benefits in modulating the bacterial flora, that are unrelated to the immediate symptoms being addressed in a low-FODMAP diet.

#### 2.2.3. Prebiotics

There is increasing evidence that the bacteria residing within the mucus layer of the colon strongly influence whether host cellular homeostasis is maintained or whether inflammatory mechanisms are triggered. These effects may either occur through direct contact with host cells, or through the production of bacterial metabolites. As well as modulating inflammation, there is also evidence that intestinal dysbiosis is involved with the development of colorectal cancer in IBD patients [62]. With increasing recognition that the gut microbiota plays an essential role in health, interest has increased in methods of modulating its composition and metabolic function. Various microbial-directed therapies have been proposed, including faecal microbial transplant, antibiotics, probiotics, and prebiotics [63,64].

One of the more attractive strategies for modulating the gut microbiota is prebiotics, which were originally defined as “selectively fermented non-digestible food ingredients or substances that specifically support the growth and/or activity of health-promoting bacteria that colonize the gastrointestinal tract” [65]. More recent arguments have been advanced, indicating that recent advances in our understanding of diet–microbiome–host interactions challenge this older concept, especially the requirement for effects to be “selective” or “specific” [66]. They propose expanding the definition and shifting the focus towards ecological and functional features of the microbiota, relevant for host physiology. Even with a wider definition, most of the materials included as prebiotics are covered in the widest definitions of dietary fibre that include the various non-digestible oligosaccharides.

Fructans as mixtures of oligofructoses and high molecular weight inulin from chicory roots have been used as prebiotic food supplements suggested for IBD. For example, Prebio 1^®^ (Nestlé, Vevey, Switzerland) and Synergy 1^®^ (Beneo-Orafti, Tienen, Belgium) contain 70% oligofructoses and 30% high molecular weight inulin. A preparation referred to as Raftilose P95^®^ (Beno Orafti, Belgium), which contains 95% oligofructoses, has also been used.

### 2.3. Dietary Fibre Intervention Studies in Inflammatory Bowel Disease

#### 2.3.1. Dietary Fibre Supplements

Of the total 23 studies of DF intervention in IBD patients, six were single-arm and 17 were placebo-controlled intervention studies, as shown in Table 1 and Table 2. DF supplements tested included fructans, psyllium, oat bran, wheat bran and germinated barley foodstuff.

##### Fructans

Lindsay *et al.* investigated the effects of supplementation with chicory fructans as Prebio 1^®^ in active CD patients in a single-arm intervention study of three weeks [40]. Ten patients received 15 g of fructans for three weeks, after which disease activity was measured using the Harvey-Bradshaw index, while other measures were assessed by fluorescence *in situ* hybridisation and flow cytometry of dissociated rectal biopsies. The fructans induced a significant reduction in the Harvey-Bradshaw index and therefore disease activity, while significantly increasing faecal *Bifidobacteria* concentration. It also increased the percentage of interleukin-10-positive dendritic cells and the percentage of dendritic cells expressing toll-like receptor 2 and toll-like receptor 4, indicating beneficial modification of mucosal dendritic cell function.

A double-blinded randomised control trial (RCT) of active CD patients taking chicory fructans as Synergy 1^®^ for four weeks showed similar outcomes to the previous study by Lindsay *et al.*, but had increased dendritic cell responses [2]. Synergy 1^®^ supplements were given for 14 days to active UC patients in a 2007 study and given for four weeks to inactive and mild-moderately active CD patients in another study in 2013 [21,42]. Casellas *et al.* found that patients recorded a reduction in dyspeptic symptoms, and the level of the faecal inflammatory biomarker, calprotectin, had decreased by day 7 of the intervention period [42]. Faecal human DNA concentrations, however, revealed no change with Synergy 1^®^ intake. The pilot double-blinded RCT with four weeks of daily intake of Synergy 1^®^ found relatively increased levels of acetaldehyde and butyrate compared to baseline in the patients [21].

Another randomised crossover study considered 16 patients (15 UC and one with familial polyposis coli), all with ileal pouch-anal anastomoses. The trial compared the effects of three seven-day supplement periods of fructans (oligofructoses as Raftilose P95^®^), RS and digestible carbohydrate intake with seven-day washout periods between them with a glucose placebo [67]. In all of these patients, fructan supplementation had a fermentation ability of 83% and was associated with an increase in faecal butyrate excretion, in comparison to RS supplementation with 46% fermentation ability and increased faecal isobutyrate and isovalerate excretion. This suggests that the fermentation ability is higher from fructan intake than from RS supplementation, and that different SCFAs can be produced depending on the type of DF.

In a trial coordinated by Joossens *et al.* (2011), faecal samples from 17 healthy volunteers who consumed 20 g of Synergy 1^®^ for four weeks, were analyzed before and after treatment [68]. DGGE fingerprinting profiles revealed that the bacterial species *Bifidobacterium longum* and *B. adolescentis* were significantly increased in faecal samples after treatment. A subsequent study by these authors considered 67 patients with inactive to mild or moderately active CD randomised to receive 10 g of Synergy 1^®^ for four weeks [69], after which they measured clinical disease activity and changes in the microbiota. Although they found no effect of the fructans on *F. prausnitzii* or *B. adolescentis*, they observed an increase in *Rhamnococcus gnavus* and *B. longum*. In those patients with active CD, the increase in this latter bacterium was associated with an improvement in the Harvey–Bradshaw index.

A case-controlled observational study (not included in the tables since no dietary changes were involved) compared the habitual intake of fructans of both inactive and active CD patients to the intake of healthy controls [70]. A total of 303 individuals were recruited, of similar numbers in the three groups, and found that patients with active CD consumed lower amounts of oligofructoses and high molecular weight fructans in comparison to the other two groups. In addition, HBI scoring revealed negative correlations of GI symptoms, in particular abdominal pain and serum CRP levels, to the total intake of dietary fructans.

Overall, these various studies investigating the effects of fructans in IBD patients suggest that this type of DF may not only relieve GI symptoms, but also increase gut immune function, reduce intestinal inflammation and beneficially modulate the GI microbiota.

##### Psyllium

Two studies by Hallert *et al.* and Fernandez-Banares *et al.* found an overall benefit in patients with inactive UC from consuming psyllium husk or seeds [33,71]. The first RCT of a four month duration found that consumption of psyllium husk improved gastrointestinal symptoms in these patients [33]. Questionnaire-based scoring of abdominal pain, diarrhoea, loose stools, urgency, bloating incomplete evacuation, mucus and constipation had improved compared to baseline. However, the 13 patients in the placebo-group also showed minor improvements, possibly because crushed crispbread that contained 17.3% of insoluble DF was given. This suggests that both supplements of psyllium husk and the “placebo” could provide benefits in reducing symptoms commonly experienced by patients. The other open-labelled RCT of 12-months duration compared the intake of psyllium seeds with or without mesalamine treatment to a control group [71]. Mesalamine is a 5-amino-salicylate with general anti-inflammatory effects, often used as front-line therapy in UC [72]. Results of this combination showed that the treatment failure rate was 40% in the psyllium group, 35% in the mesalamine group and 30% in the group taking psyllium and mesalamine together. However, these differences were not statistically significant, and the probability of continued remission was similar in all three groups. Furthermore, patients consuming psyllium seeds had increased faecal levels of butyrate, which the authors linked to the maintenance of disease remission.

A randomised crossover study investigated the intake of wheat bran and psyllium in two six-month intervention periods, with a six-month washout period using a placebo of molded crisps between (we note that the nature of the crisps was not defined in the original paper) [73]. Ejderhamn *et al.* showed that there were differences between the two DF supplements in children with inactive UC. Unlike wheat bran, psyllium supplement did not significantly change bile acid measurements in these patients. Bile acids are critical for the digestion and absorption of fats and fat-soluble vitamins in the small intestine, and may indirectly help to shape the gut microbiota [74]. This study showed that wheat bran intake is more effective in reducing bile acid concentration than a psyllium supplement in an inactive UC condition.

##### Oat Bran

A 12-month pilot RCT investigated the intake of oat bran, as a source of 1,3;1,4-β-glucans, in patients with inactive UC. This trial showed a 30% increase in the concentration of faecal butyrate and improvements in abdominal pain or gastroesophageal reflux after oat bran intervention [37]. Moreover, this intervention caused no increase in colitis relapse nor GI complaints.

##### Wheat Bran

An interview-based single-arm study of wheat bran (WB) intake for four weeks found that CD patients perceived symptomatic benefits that included reductions in diarrhoea, pain and cramps, urgency and incontinence, and borborygmus (stomach gurgling) [75]. This suggests that increased intake of wheat bran in CD patients may reduce the severity of GI symptoms.

As previously discussed, Ejderhamn *et al.* performed a randomised crossover study to compare the intake of WB and psyllium in two six-month intervention periods, with a six-month washout period [73]. WB intake was associated with significant reductions in faecal bile acid concentrations. Bile acids are critical for the digestion and absorption of fats and fat-soluble vitamins in the small intestine, and may indirectly help to shape the gut microbiota [74]. However, the reduction in specific bile acids shown to be affected in this study may be beneficial to the patients, since they have been reported to induce a proliferative response in the gastric mucosa in rats, and may also increase mucosal cell proliferation in other parts of the GI tract [73].

Although no specific supplements were prescribed in their intervention study, Brotherton *et al.* provided a half cup of wheat bran cereal per day, and general instructions for reducing sugar intake while maintaining fluid intake [4]. The authors demonstrated that consuming this high-DF and low refined carbohydrate intervention was feasible in CD patients during a four-week RCT [4]. No adverse effects were associated with this diet which was shown to improve the GI function and quality of life (as measured by Harvey–Bradshaw index) in these patients.

##### Germinated Barley Foodstuff

A pilot single-arm study with germinated barley foodstuff supplementation (containing 34% DF) in active UC patients showed clinical and endoscopic improvements following intervention for four weeks [34]. Furthermore, stool butyrate concentrations had increased in these patients. An open-controlled RCT was also conducted with the same supplement, for the same time, in active UC patients [36]. They found a reduction in clinical activity index scores in these patients compared with the starting value, and this was associated with an increase in faecal concentrations of *Bifidobacterium* and *Eubacterium limosum*. In addition to these studies, a more recent open-labelled RCT with a longer duration of two months was conducted in 2014 by Faghfoori *et al.* [76]. This group tested germinated barley foodstuff supplementation in patients with inactive UC and found a decreased mean serum level of CRP, along with symptomatic improvements in reducing abdominal pain and cramping. Overall from these three studies, germinated barley foodstuff supplementation in UC patients leads to improvements in GI symptoms and clinical disease activity that could be caused by elevated proportions of beneficial microbes produced in the gut.

#### 2.3.2. Diets with Altered Dietary Fibre Content

Specific dietary interventions that modified DF content included a low-FODMAP diet, which lowers certain sugars and also NDOs; a low-DF diet; a DF-rich, unrefined carbohydrate diet; a semi-vegetarian diet (SVD); and diets containing a high *versus* low ratio of wheat bran to high amylose-associated resistant starch (WB:RS).

The low-FODMAP diet has been used in irritable bowel syndrome with some apparent symptomatic benefits, and has been suggested also for IBD. Two single-arm intervention studies lasting six weeks and three months, respectively, investigated a low-FODMAP diet, mainly in patients with IBD and one with chronic constipation [77,78]. The shorter trial showed an overall reduction in stool frequency in patients without pouchitis, whereas the longer trial showed improvements in abdominal symptoms in pain, bloating, wind and diarrhoea, but not in constipation. The authors suggested that the low-FODMAP diet previously tested in irritable bowel syndrome patients may also prove to be beneficial in relieving some IBD symptoms. It is important to note, however, that such an intervention lowers NDOs. The trials did not use a quantitative endpoint of disease activity, such as the Harvey-Bradshaw index, nor did they consider changes of faecal microbiota, both of which have been beneficially associated with increased NDO consumption.

A DF-rich, unrefined carbohydrate diet was used as an intervention in CD patients by Heaton *et al.* and Jones *et al.* [30,32]. The single-arm trial by Heaton *et al.* of duration ranging 18–80 months, found a favourable effect in these patients [30]. The intervention was associated with fewer and shorter hospital admissions, 111 days compared with matched controls of 533 days. Moreover, this diet did not trigger intestinal obstruction as only one patient on the intervention required surgery compared with five of the control patients. It should be noted, however, that this trial has been criticised because controls were historic.

A 29-month RCT compared a low-DF diet intervention with a normal Italian diet in 70 patients with non-stenosing CD [79]. The study had been designed because Italian CD patients were regularly being advised to follow a low-DF diet. The low-DF patients were advised against legumes, whole grains, nuts, and whole fruits and vegetables, except for ripe bananas and skinned potatoes, while the normal Italian diet included liberal fruit and vegetable intake. Results from this study showed no statistically significant difference in clinical disease activity and symptomatic scoring between the two dietary groups, although there were non-significant improvements in disease recurrence in the control group, following the normal Italian diet. We would note, however, that it is not just DF content that will have changed by this dietary regime.

Jones and co-workers designed an RCT in which a DF-rich, unrefined carbohydrate diet was compared with an exclusion diet, individually tailored to the patient [32]. We have shown that such exclusion diets recognise genetic differences and enable very precise definition of optimal diet for each patient [24]. Jones *et al.* showed a worse outcome in active CD patients taking the DF-rich, unrefined carbohydrate diet for six months. After a six-month intervention, seven of the 10 patients on an exclusion diet maintained disease remission, compared with none on the DF-rich, unrefined carbohydrate diet) [32].

A two intervention-period crossover study in IBD patients who had undergone colectomy compared for two weeks each the ileostomy fluid output after a Western diet of refined cereal food intake (diet A) with a diet containing increased amounts of unrefined cereal foods (diet B) [31]. Diet B was found to be more effective in producing a greater ileostomy effluent output and increased bacteriological flora/gram as compared with diet A.

In a randomised control trial, inactive CD patients eating a DF-rich Japanese semi-vegetarian diet were shown to maintain disease remission with a rate of 100% at the end of year-1 and 92% at the end of year-2 [41]. Nine of the patients consuming the semi-vegetarian diet who had maintained remission (60%) had normal CRP concentrations. The authors suggested the semi-vegetarian diet could more effectively prevent CD relapse as compared to an omnivorous diet, for which 33% maintained remission over this time.

A two-period crossover study (two weeks on each arm with a two-week washout period) investigated the outcomes of patients with inactive UC, randomly assigned either “low RS/WB” foods (2–5 g RS and 2–5 g WB fibre per day), or “high RS/WB” foods containing 15 g RS and 12 g WB fibre per day [43]. The RS (Types 1 and 2) was present in coarsely ground high-amylose maize, whereas the WB was a combination of processed twig cereal and unprocessed WB. Compared with healthy controls, the UC patients had a different microbiota structure with lower proportions of *Akkermansia muciniphila*, and a greater diversity of *Clostridium* cluster XIVa species. These patients also had a reduced ability to ferment DF, possibly related to the altered microbiota, and they also had variable gut transit times. Even eating the diet high in RS and WB did not increase the proportion of carbohydrates fermented or the production of faecal short-chain fatty acids, at least over the two-week intervention period. However, eating this diet did normalize the gut transit times. The diet high in RS and WB may thus have some benefits for inactive UC patients.

## 3. Discussion

### 3.1. Is Dietary Fibre Beneficial to IBD Patients?

The studies summarised above make it clear that a single generalisation as to the benefits or otherwise of increasing DF in IBD cannot be made. This is not surprising, given that DF is not a homogeneous material, but represents a complex group of chemicals with differing properties. There are numerous other factors that will have influenced the outcomes from DF intervention in the current studies. Not only the type of fibre but also the dosage and length of time over which the DF intake occurred are likely to be important. The results are also likely to be affected by whether the patients had UC or CD, whether the disease was in remission or not and whether they had an intact colon or not. It is thus not surprising that DF failed to produce consistent symptomatic relief and disease remission in patients suffering from IBD.

This same heterogeneity in DF source and study design also makes it very difficult to evaluate the relative quality of the studies done. No two studies used the exact same DF and control treatment, and the study numbers would not meet current international criteria for design of an epidemiological or dietary intervention study. We recognise this as a limitation of the current review. Nevertheless, some clear patterns emerge.

Early studies with diets described as DF-rich, unrefined-carbohydrate diets gave apparently different results [30,32]. Whereas Heaton *et al.* reported that this had favourable effects in CD [30], Jones *et al.* found such a diet inferior to an exclusion diet in which IBD patients excluded specific foods that they had found to exacerbate their GI symptoms [32]. However, more recent work has shown the importance of genotyping in interpreting dietary tolerances and intolerances [23,24]. The apparent differences between the two sets of study results may mean that, despite using the same diet as used by Jones *et al.* Heaton *et al.* have selected a different set of patients, who may have had different genetic susceptibilities [30]. Unfortunately, this question was not asked at the time of those studies.

The low-residue diet used in the RCT of CD patients had no significantly different outcomes as compared with that of the normal Italian diet [79]. As these patients had limited DF (mainly in the form of insoluble DFs) to <10–15 g/day in their diets, this might be taken to imply that reducing DF intake would have little effect in IBD [3]. However, the data may alternatively be interpreted to suggest that a normal Italian diet, which is rich in plant foods and DF, had no detrimental effects on the disease, and the subjects could now go on to try diets containing even higher amounts of DF.

The amount of DF supplementation consumed by patients daily could also contribute to the outcomes in the studies. Other than a given dosage of DF in some studies, dietary advice was provided to patients regarding recommended food sources for increasing DF consumption. Moreover, the tolerance to interventions would be reliant on the dosage of DF. In these studies, tolerance was generally calculated from recollecting unused sachets after the intervention period [2,71,73]. Comparisons of DF compliance between the intervention group and placebo group would determine the feasibility of whether such interventions can be carried out long term and may alter both beneficial and adverse effects. It is also likely that in some studies, compliance in taking DF supplements was higher than in other studies.

Another concern that arises from human intervention studies is the possibility that the supplement given as placebo in RCTs can additionally contain some type of DF. Most likely this unaddressed problem would lead to erroneous conclusions from the data collected in the clinical trial. One noticeable study conducted by Hallert *et al.* (1991) had a placebo of crispbread, which contained 17.3% insoluble DF [33]. Although the effective psyllium supplementation contained higher amounts of DF, the placebo also proved to be somewhat effective in reducing GI symptoms as compared to baseline.

What is known about intervention with DF is that at least some of these when consumed have the potential to relieve symptoms and/or to induce and maintain disease remission in IBD patients through mediating various GI processes. However, the exact result seen could rely on all the above factors that can modify the extent of benefits. It is apparent that certain types of DF in certain patients, at certain times, can increase the severity of some adverse events such as elevated incidence of flatulence and “gut rumbling” [40] and/or further exacerbate other disease symptoms (which caused a few patients undergoing DF intervention to withdraw from the clinical trials). We note that this may be a side effect of many fructans in particular, and may be the main benefit of the low-FODMAP diet. However, although such direct effects are inconvenient to the patient, they do not associate with long-term detrimental effects. It is unfortunate that intestinal eubiosis/dysbiosis was not considered as an informative endpoint in many of the trials.

Other factors that could have an influence on the outcomes of the studies could be the concurrent patient’s diet. Some studies such as the trial done by Benjamin *et al.* and Kanauchi *et al.* did not recognise the importance of genotyping and did not restrict or advise against foods that could be detrimental for the IBD condition, therefore potentially masking the beneficial effects of intervention with DFs [2,36].

### 3.2. Possible Mechanisms of Action of Beneficial Dietary Fibres in IBD

**Figure 1:**
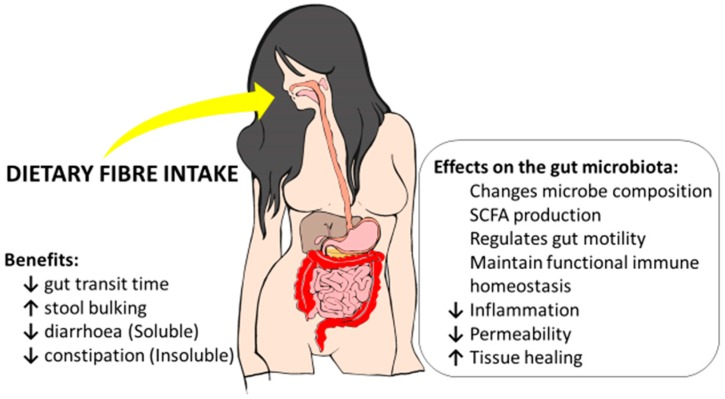
A summary of potential benefits, including effects on the gut microbiota, for inflammatory bowel disease from increased dietary fibre intake.

As discussed above, the evidence indicates that increasing DF may often be beneficial in IBD patients. It becomes important to distinguish between sources of DFs rather than DFs *per se*. For example, diets enriched in food plants (e.g., semi-vegetarian diets) will increase the proportion of a range of different types of DFs in the diet. Direct action on the immune system may be important for some types of DF. Directing the therapeutic objective of DF intervention to aid in the modification of the microbiota composition in the inflamed gastrointestinal tract in IBD patients may indirectly improve its immunologic function and modify the disease course over the long term [80,81,82]. Certain fructans acting as prebiotics may be important in this respect. Furthermore, other outcomes associated with other types of DF may aid in diminishing the risk for subsequent clinical relapses [83,84]. Many of the potential mechanisms are summarised in Figure 1.

In healthy individuals, more than 90% of the gut bacterial population are members of two phylla: Firmicutes and Bacteriodetes [85]. However in IBD patients exhibiting chronic inflammation in the gut, this microbial composition is altered and the presence of potentially pathogenic microorganisms tends to be a prominent contributor of intestinal dysbiosis [86]. Links have been postulated between the intestinal dysbiosis that occurs in IBD and inflammatory processes, but whether one factor is a consequence of the other is unclear [87]. Diet and genetic susceptibility are also known to influence the development of this condition [23].

Certain bacterial species, including *Bifidobacteria* and *Faecalibacterium prausnitzii*, have been shown to cause immunoregulatory effects following activation of dendritic cells that recognise bacteria in the intestinal microbiota, using pattern recognition receptors such as toll-like receptors (TLRs) that bind to distinct surface molecules (pathogen-associated molecular patterns or PAMPs) [40,88,89]. As a result, bacterial secretion of cytokines stimulates corresponding T-cell responses such as Th1 (pro-inflammatory), Th2 or Treg (both anti-inflammatory) pathways [90]. It is evident that *Bifidobacteria* activity can cause an increase in DC IL-10 stimulated release and reduce interferon-γ (IFN-γ) production by activated CD4^+^ T cells [91] whereas *Faecalibacterium prausnitzii* can increase IL-10 production and decrease peripheral blood IL-12 levels, at least in human *in vitro* experiments [92]. Furthermore, the presence of *F. prausnitzii* has been correlated to protecting patients from IBD and a higher proportion of this bacterium in the gut microbiota tends to associate with disease remission [92,93].

Another difference observed in patients with intestinal inflammation compared with healthy individuals is an impaired intestinal barrier thought to be controlled by diet and bacterial species residing in the gut microbiota [94]. An increased permeability of the tight junctions of the gut epithelium results in the dysregulated absorption of nutrients and toxins. A physically intact and immune-functional intestinal barrier that protects the host from pathogenic invasion is disrupted in IBD. This may disturb the gut mucosa and perpetuate inflammatory processes. By down-regulating inflammation and restoring the integrity and homeostasis of the intestine, immune responses against consumed pathogens could be theoretically strengthened [11]. One possible method of fortifying protection for IBD can be through the intake of specific types of DF which can enhance the growth and activity of beneficial microbes. Various prebiotic DFs have been found to have this activity [95].

DF polysaccharides provide a crucial energy source for bacterial activity in the large intestinal microbiota, which can directly or indirectly influence gut mucosal immune responses [96]. A mechanistic effect referred to in many of the DF studies is the bacterial fermentation of certain DF polysaccharides in the colon by beneficial gut microbes, including *Bifidobacterium* [2,40], *Clostridium* cluster XIVa species [43], and *Eubacterium limosum* [36], with the production of SCFAs including butyrate [21,34,37,67,71]. Other SCFAs produced by the gut microbiota include acetate, propionate and valerate [90]. SCFAs act as a fuel source for colonocytes and have been suggested to modulate inflammation in the intestine by enhancing the proportion of non-pathogenic microbes in the gut microbiota [97]. Butyrate has been shown to regulate a number of processes that involve fluid transport through the gut epithelium, ameliorate gut inflammation and oxidative stress, mediate immune regulation, and modulate intestinal motility. Butyrate can exert anti-inflammatory and anti-carcinogenic effects to the host through epigenetic modifications associated with the inhibition of histone deacetylases [98,99]. In addition, butyrate can alter the maturation of dendritic cells and induce the production of anti-inflammatory cytokines that favour dendritic cell-secretion of IL-10 and its activity *in vitro* [100]. Thus immunomodulation of the mucosal gastrointestinal tract may aid in alleviating IBD activity and maintain disease remission.

From the discussion above, it is apparent that fructans have beneficial effects in IBD. However, the low-FODMAP diet which reduces or eliminates such molecules is claimed to have produced favourable outcomes for IBD patients [61,101]. A critical evaluation of the studies purporting to support a low-FODMAP diet, however, shows that the endpoints have largely involved short-term measures of discomfort such as gas and bloating, rather than considering longer term effects on intestinal dysbiosis or inflammatory measures. For these reasons, we would caution against the acceptance of such a regime.

Psyllium supplementation was indicated to be as effective as drug treatment with mesalamine [71]. Psyllium is known for its effects on bowel regularity, and indeed is sold over the counter as a natural laxative [102]. This material consists of a mixture of insoluble and soluble fibres, which may have different effects. However, there is reason to believe that the active component here is the soluble, gel-like component, which is known to be a complex highly substituted heteroxylan [54].

Wheat bran is known to consist of ~46% non-starch polysaccharides (NSP) from arabinoxylans, cellulose, and 1,3;1,4-β-glucans [103]. Its use appears beneficial in IBD patients, although not as a replacement for medication [104]. We have also reported that wheat bran is able to protect against colon cancer [28,29]. Given the excess risk of this type of cancer in IBD patients, the regular use of wheat bran might appear to be beneficial.

It is also worth considering that non-DF components of DF-enhanced diets may be active. Even when the diet is supplemented with products actually enriched in DFs, other components may still play at least some role. An example here might be the glutamine-rich protein in the GBF. Although GBF is a rich source of DF, a major component is glutamine-rich protein (46%), and glutamine is known to enhance the growth and repair of the gut mucosa [11]. Considering this, it is possible that the interaction between this amino acid and DF can create synergistic effects that are effective in relieving GI-associated symptoms in IBD patients. Wheat bran also has a number of associated phytochemicals, which may augment the effects of the DF [105].

## 4. Materials and Methods

A literature search was performed using the medical journal databases: MEDLINE, Scopus, Web of Science and PubMed, with no time period restriction. Keywords included: Inflammatory bowel disease, Crohn’s, or ulcerative colitis, in association with dietary fibre, dietary fiber, fibre types, fructo-oligosaccharide, galacto-oligosaccharide, non-digestible oligosaccharide, fructan, resistant starch or oligofructose. The search was limited to the English language.

Accessible intervention studies were selected involving IBD patients that were either treated with or professionally advised to take single supplements, or diets enriched in specific dietary fibres. The studies included from 1979–2015 were of variable study designs, such as pilot randomised control studies (RCTs), double-blinded RCTs, and crossover studies, that had targeted DF interventions for IBD. Sample sizes of the intervention studies ranged from 7 to 103.

## 5. Conclusions

Most of the intervention studies have used dietary fibre (DF) supplements that were not a single fibre type, but rather mixtures of different types of DF. Whether the disease is inactive or active and its degree of activity could also contribute to the differences seen in the outcomes of these studies. It would seem unlikely that DF intervention alone would prove effective in maintaining disease remission, and indeed an early study that compared medication with a DF intervention showed the latter to be inferior [104]. However, as discussed above, certain types of DF in conjunction with medication would appear appropriate in helping to control disease symptoms. Other types of DF may also be important in controlling the progression of inflammatory bowel disease (IBD) to colorectal cancer. In conclusion, researchers using dietary interventions need to be wary that there are considerable differences in associated gastrointestinal tract (GIT) effects, due to whether the DF is obtained from whole foods or is given in an altered supplement form that has been isolated or extracted from its original food source. It is also essential to distinguish specific DFs. Therefore, professional recommendations for IBD patients should be directed towards utilising the intake of specific DF types as discussed above.

## Figures and Tables

**Table 1:** Single-arm intervention studies involving dietary fibre.

Subjects	Fibre Source(s), Dosage, Trial Duration	Number/Groups	Measured Endpoints	Results
Active CD patients [40]	Chicory fructans as Prebio 1^®^ (Nestlé, Vevey, Switzerland), 15 g/day, 3 weeks	Total = 10	HBI, CDAI scores, serum CRP, full blood count, faecal and mucosal biopsy measurements	↑ faecal *Bifidobacterium*
				↑ IL-10 released by intestinal dendritic cells
				↓ disease activity
Healthy volunteers [68]	Oligofructose-enriched inulin (Synergy 1^®^), 10 g/2 times daily, 4 weeks	Total = 17	Faecal sampling	↑ *Bifidobacterium longum* counts
				↑ *Bifidobacterium adolescentis* counts
Inactive CD patients [75]	Wheat bran, NA, 4 weeks	Total = 11	4 semi-structured audio-recorded interviews	Experienced benefits: ↓ diarrhoea, pain/cramps, urgency and incontinence, and borborygmus (stomach gurgling)
Active UC patients [34]	Germinated barley foodstuff, 30 g/3 times daily, Pilot 4 weeks	Total = 10	CAI score, endoscopic index, serum CRP, ESR, and stool SCFA measurements	Clinical and endoscopic improvements
				↑ stool butyrate concentrations
Inactive IBD patients [77]	Low FODMAP diet (Specific DFs (NDOs) reduced), NA, Pilot 6 weeks	Total = 15	Carbohydrate malabsorption breath testing, pouchitis assessed either clinically or endoscopically, faecal lactoferrin, and 7-day food diary	↓ Short-term overall stool frequency in patients without pouchitis
		UC = 13		
		CD = 1		
		Chronic Constipation = 1		
Inactive UC and CD patients [78]	Low FODMAP diet (Specific DFs (NDOs) reduced), NA, Pilot 3 months	Total = 72	Telephone questionnaire and interview	Short-term improvements in abdominal symptoms: ↓ pain, bloating, wind and diarrhoea
		CD patients = 52		Constipation did not significantly improve
		UC patients = 20		
Active CD patients [30]	DF-rich, unrefined-carbohydrate diet, NA, 18–80 months	Total = 32	Postal questionnaire, clinical hospital admissions and surgery frequency (historic control)	Favourable effect: ↓ numbers and duration of hospital admissions in the diet treatment (111 days) compared with matched controls (533 days)
				Did not cause intestinal obstruction: 5 controls required surgery *vs.* 1 patient in the diet treatment

NA: not applicable; CAI: clinical activity index.

**Table 2:** Placebo-controlled intervention studies involving dietary fibre.

Human Subjects	Fibre Source(s), Dosage, Trial Type/Duration	Number/Groups	Measured Endpoints	Results
Active CD patients [2]	Chicory fructan as Synergy 1 (Beneo Orafti, Belgium), 15 g/day, RCT (DB)/4 weeks	Total = 103	CDAI, IBDQ, serum CRP, ESR, platelet count, and faecal calprotectin measurements	↓ disease activity
		Fructan = 54		↑ faecal bifidobacteria counts
		Placebo with maltodextrin = 49		↑ dendritic cell responses
Inactive and mild-moderately active CD patients [21]	Chicory fructan as Synergy 1 (Beneo Orafti, Tienen, Belgium), 10 g/2 times daily, Pilot RCT (DB)/4 weeks	Total = 56	HBI, and faecal sampling	↑ relative acetaldehyde and butyrate levels
		Fructan = 31		
		Placebo = 25		
Active UC patients [42]	Chicory fructan as Synergy 1 (Beneo Orafti, Belgium), 4 g/3 times daily, Pilot RCT/14 days	Total = 19	Rachmilewitz score for dyspeptic symptoms, faecal calprotectin and faecal human DNA measurements	Synergy 1 well tolerated
				↓ in dyspeptic symptoms
		Fructan = 10		↓ calprotectin at day 7
		Placebo with maltodextrin = 9		No change in faecal human DNA concentration
UC patients with ileal pouch [67]	Chicory fructans (Raftilose P95^®^, Beneo Orafti, Belgium) placebo with glucose, 14.3 g daily, 3-period crossover/three 7-day supplement periods with 7-day washout periods	Total = 15	Faecal and breath sampling, self-reported diary record	Fructan supplementation: Fermentation ability is 83% ↑ faecal butyrate excretion
				RS supplementation: Fermentation ability is 46% ↑ faecal isobutyrate and isovalerate excretion
Inactive and active CD patients [69]	Chicory fructan as Sygergy 1), 10 g/2 times daily, RCT (DB)/4 weeks	Total = 45	HBI, and faecal sampling	↓ faecal *Ruminococcus gnavus* counts
		Fructan = 25		↑ faecal *Bifidobacterium longum* counts
		Placebo = 20		↓ disease activity in active CD patients
				No effect on *F prausnitzii*
Inactive UC patients [33]	Psyllium husk (Vi-Siblin S^®^, Parke-Davis), 3.52 g daily, RCT/4 months	Total = 29	Questionnaire, a visual analogue scale	Diet is proven safe and improves gastrointestinal symptoms: of abdominal pain, diarrhoea, loose stools, urgency, bloating, incomplete evacuation, mucus and constipation
		Psyllium husk = 16		
		Placebo with crushed crispbread = 13		
Inactive UC patients [71]	Psyllium seeds (including husk), combined with mesalamine, and placebo of mesalamine alone, 10 g psyllium sachets—2 times/day and 500 mg drug tablets—3 times/day, Open-label RCT/12 months	Total = 102	Standardised questionnaire and examination, haematological, biochemical and urine measurements, a daily symptomatic diary, and a sigmoidoscopic analysis	Both failure rate and continued remission of similar approximations: 40% in psyllium; 35% in mesalamine; 30% in psyllium + mesalamine
		Psyllium = 35		
		Mesalamine only = 37		
		Psyllium plus mesalamine = 30		↑ faecal butyrate levels in psyllium
Inactive UC children [73]	Wheat bran (processed) (Fiber-form^®^), Psyllium husk (Lunelax^®^) and placebo with molded crisps, 3.5 g DF sachets daily, Crossover/two 6-month intervention periods with a 6-month washout period between	Total = 10	Faeces sampling, diary record, and Talstad & Gjone clinical disease activity scoring	WB supplementation: ↓ faecal bile acid concentration (by 43%) ↓ faecal water concentration of bile acid (by 55%)
				Psyllium supplementation: Did not ↓ faecal bile acid or water concentrations
Inactive UC patients [37]	Oat bran as source of 1,3;1,4-β-glucans, 60 g of oat bran (20 g of 1,3;1,4-β-glucans, Pilot RCT/12 weeks	Total = 32	Every 4-weeks clinical assessments, stool samples, Seo activity index, and GSRS questionnaire	↑ by 30% of butyrate concentrations in faeces at week 4
				No signs of an increase in colitis relapse
		Oat bran = 22		No ↑ in gastrointestinal complaints
		Control = 10		At entry, improvements of abdominal pain or gastroesophageal reflux
Inactive CD patients [4]	DF-rich, unrefined carbohydrate diet (including wheat bran), NA, RCT/4 weeks	Total = 7	IBDQ, pHBI, telephone interview, serum CRP, and ESR measurements	Diet consumption was feasible
		High-fibre diet = 4		No adverse effects
				Improved quality of life and gastrointestinal function
				No significant difference between groups in the inflammatory biomarkers
		Control diet = 3		
Active UC patients [36]	Germinated barley foodstuff, 20–30 g daily, Open-control RCT/4 weeks	Total = 18	CAI score, colonoscopic examination, faecal and blood samples	↓ clinical activity index scores
		GBF = 11		↑ faecal *Bifidobacterium* and *Eubacterium limosum* concentrations
		Control with anti-inflammatory treatment = 7		
Inactive UC patients [76]	Germinated barley foodstuff, 30 g/3 times a day, Open-labelled RCT/2 months	Total = 46	Serum CRP level, and clinical oral assessment	↓ mean serum CRP
		GBF = 23		Symptomatic improvements: ↓ abdominal pain and cramping
		Control with conventional medication only = 23		
Active CD patients [32]	DF-rich, unrefined-carbohydrate diet *vs.* exclusion diet, NA, RCT/6 months	Total = 20	Time to disease remission	UCFR diet: None remained in disease remission
		UCFR diet = 10		
		Exclusion diet = 10		Exclusion diet: 7/10 in remission for 6 months
Inactive and active CD patients [79]	Low-residue (*i.e.*, DF) diet *vs.* normal Italian diet, NA, RCT/a mean of 29 months	Total = 70 (58 active CD, 12 inactive CD)	CDAI, 5-point scale rating pain and diarrhoea, and interview	No significant difference in outcome between the 2 diet groups
		Low-residue diet = 35		
		Normal Italian diet = 35		
CD and UC patients who had undergone colectomy [31]	Diet A (Western diet of refined cereal food intake) *vs.* Diet B (increased unrefined cereal food intake), NA, Crossover/two 2-week intervention periods with 1-week rest period between	Total = 10	Ileostomy fluid output	Effects of diet B (compared to diet A): ↑ ileostomy effluent amount (both wet and dry weight) ↑ bacteriological flora/gram
		CD patients = 5		
		UC patients = 5		
Inactive CD patients [41]	Semi-vegetarian diet, NA, RCT/2 years	Total = 22	Kaplan-Meier survival analysis, and serum CRP measurement	Remission was maintained: Remission rate of 100% at year 1 and 92% at year 2 follow-up; 9 (of 15) SVD patients who maintained remission had normal CRP concentrations at the end of trial
		SVD = 16		
		Omnivorous control = 6		
Inactive UC patients [43]	Wheat bran (45% DF) and coarsely ground high-amylose maize HiMaize^®^ as source of 30% RS (Types 1 and 2), High RS/wheat bran (15 g RS plus 12 g wheat bran DF/daily) *vs.* Low RS/wheat bran (2–5 g RS plus 2–5 g wheat bran DF/daily), Crossover/two 17-day intervention periods with a 14-day washout period between	Total = 29	Faecal output, whole gut transit time measurement, food diary, CAI, and 4-point Likert scale	In UC patients (than control): ↑ 3-fold in faecal NSP and starch concentrations
		UC patients = 19		
		Healthy control = 10		High-RS/WB intake in UC patients: Normalised gut transit; Change in gut bacterial composition (low count of *Akkermansia muciniphila*, and greater diversity of *Clostridium* cluster XIVa species)

## Abbreviations

CAI	clinical activity index
CD	Crohn’s disease
CDAI	Crohn’s disease activity index
GIT	gastrointestinal tract
CRP	C-reactive protein
DB	double-blind
DF	dietary fibre
ESR	erythrocyte sedimentation rate
GSRS	gastrointestinal symptom rating scale
HBI	Harvey–Bradshaw Index
IBD	inflammatory bowel disease
IBDQ	inflammatory bowel disease questionnaire
IL-10	interleukin-10
pHBI	partial Harvey-Bradshaw Index
RCT	randomised control trial
RS	resistant starch
SVD	semi-vegetarian diet
SA	single-arm intervention
SCFA	short-chain fatty acid
UC	ulcerative colitis
